# Daily activity profiles over the lifespan of female medflies as biomarkers of aging and longevity

**DOI:** 10.1111/acel.14080

**Published:** 2024-01-24

**Authors:** Han Chen, Hans‐Georg Müller, Vasilis G. Rodovitis, Nikos T. Papadopoulos, James R. Carey

**Affiliations:** ^1^ Department of Statistics University of California at Davis Davis California USA; ^2^ Department of Agriculture Crop Production and Rural Environment University of Thessaly Volos Greece; ^3^ Department of Entomology University of California at Davis Davis California USA

**Keywords:** biomarker, daily activity profile, functional data analysis, lifespan, longevity, medflies, repeated functional data

## Abstract

The relationship between the early‐age activity of Mediterranean fruit flies (medflies) or other fruit flies and their lifespan has not been much studied, in contrast to the connections between lifespan and diet, sexual signaling, and reproduction. The objective of this study is to assess intra‐day and day‐to‐day activity profiles of female Mediterranean fruit flies and their role as biomarker of longevity as well as to explore the relationships between these activity profiles, diet, and age‐at‐death throughout the lifespan. We use advanced statistical methods from functional data analysis (FDA). Three distinct patterns of activity variations in early‐age activity profiles can be distinguished. A low‐caloric diet is associated with a delayed activity peak, while a high‐caloric diet is linked with an earlier activity peak. We find that age‐at‐death of individual medflies is connected to their activity profiles in early life. An increased risk of mortality is associated with increased activity in early age, as well as with a higher contrast between daytime and nighttime activity. Conversely, medflies are more likely to have a longer lifespan when they are fed a medium‐caloric diet and when their daily activity is more evenly distributed across the early‐age span and between daytime and nighttime. The before‐death activity profile of medflies displays two characteristic before‐death patterns, where one pattern is characterized by slowly declining daily activity and the other by a sudden decline in activity that is followed by death.

## Graphical abstract

The objective of this study is to assess intraday and day‐to‐day activity profiles of female medflies and their role as a biomarker of longevity as well as to explore the relationship between these activity profiles, diet, and age‐at‐death. The result shows that an increased risk of mortality is associated with increased activity in early age, as well as with a higher contrast between daytime and nighttime activity.

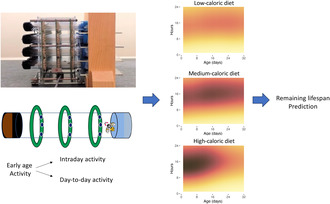

**Abbreviations**
C‐10: 10% sugar and yeast hydrolysate diet
C‐20: 20% sugar and yeast hydrolysate diet
C‐50: 50% sugar and yeast hydrolysate diet
FDA: functional data analysis
FPCA: functional principal component analysis
Medfly: Mediterranean fruit fly (Ceratitis capitata)

## 1 INTRODUCTION

Model organisms, such as fruit flies, are non‐human species which are commonly used in research to investigate biological phenomena and parameters regarding life history traits of vertebrates and humans (Leonelli & Ankeny, 2013). Fruit flies, especially the common fruit fly Drosophila melanogaster (Diptera: Drosophilidae) and the Mediterranean fruit fly (medfly) Ceratitis capitata (Wiedemann) (Diptera: Tephritidae), have regularly been used as model organisms in aging and behavioral research (Carey et al., 2006; Gargano et al., 2005; Markowska & Breckler, 1999; Promislow et al., 2022). Common fruit fly behaviors include general activity (Prenter et al., 2013; Zou et al., 2011) and specific behaviors such as sexual signaling (Zhang et al., 2006), supine behavior (Papadopoulos et al., 2002), feeding (Fanson et al., 2013), resting (Chiu et al., 2013) and sleeping (Koh et al., 2006). These behaviors are known to vary during the life course of individuals, exhibiting age‐related patterns (Carey et al., 2006).

The study of the mortality and longevity of fruit flies is well established in the biodemographic literature. For instance, Zhang et al. (2006) provided evidence that high levels of recent calling activity in male medflies are linked to longer remaining lifespans. Müller et al. (2001) demonstrated that the rate of exponential decrease in egg laying by female medflies is predictive for their remaining lifespan, while Harshman and Zera (2007) investigated the connection between early‐life increments in reproduction and later survival and explored the biological mechanisms behind this relationship.

Changes in spontaneous activity patterns are usually correlated with environmental conditions and food quality in the field (Hendrichs et al., 1991; Promislow et al., 2022) and have been considered in the past as biomarkers of “functional senescence” (Jacobson et al., 2010), providing insights to understanding the aging process of living organisms (Markowska & Breckler, 1999; Simon et al., 2006). Kaladchibachi et al. (2019) analyzed the circadian patterns of activity change in female Drosophila ananassae and studied its relationship with aging. Although previous studies aimed at investigating this relationship, they have been limited in their methodology and did not feature continuous comprehensive monitoring (Le Bourg, 1987; Lints et al., 1984; Sohal, 1985; Sohal & Buchan, 1981). For instance, Sohal and Buchan (1981) stated that the lifespan of individual flies corresponds to their levels of physical activity observed between day 4 and day 7, while Le Bourg (1987) found no significant correlation between lifespan and average locomotor activity observed once per week. However, these studies used an aggregated activity score as a measure of activity, which does not provide a comprehensive quantification of the daily activity profile. Furthermore, none of these studies monitored activity longitudinally with a 24/7 activity monitor throughout the lifetime of each individual, which provides a more accurate and detailed representation of activity and thus the relationship between movement activity levels and lifespan. Additionally, the correlation calculation used in these studies was not amenable to the exploration of more complex dependencies between lifespan and activity levels.

In our study, we used the advanced LAM25 system to obtain repeated observations of the 24‐h movement activity of medflies. The system records the movement of each fly as it crosses a barrier. We model the intra‐day and day‐to‐day activity profiles and their relationship with the remaining lifespan of each individual through functional data analysis (FDA) for repeated observations. This approach models the activity profiles as manifestations of an underlying function‐valued stochastic process (Chen et al., 2017; Chen & Müller, 2012; Wang et al., 2016). In our analysis, the longitudinal time is the age of the fly (in days) and the repeated observations consist of the daily activity records that are recorded for each day (0–24 h). By addressing the limitations of prior research, our approach leads us to hypothesize that activity profiles may serve as effective biomarkers for assessing the aging and longevity of medflies.

The goals of our investigation are (1) To study the relationship between activity and aging dynamics and the role of activity as a biomarker of longevity, for which we utilize function‐valued principal component analysis (Chen & Müller, 2012) for repeated functional data to construct the principal components of the underlying activity process, separating the intra‐day and inter‐day components. (2) To investigate the connection between early‐age activity and lifespan at the subject level. For this we use functional linear regression to predict an individual's remaining lifespan, given the activity profile and diet. (3) To explore before‐death patterns of decline in activity.

## 2 DATA DESCRIPTION

The protocol of Rodovitis et al. (2022) was followed for both measuring detailed locomotor activity and maintaining the flies in the glass tubes. The day following adult emergence, we placed virgin females of the Mediterranean fruit fly laboratory strain “Benakio,” which was maintained under laboratory conditions for more than 30 years (Kyritsis et al. (2019)), in the tubes of the locomotory activity Monitor‐LAM25 system and maintained them until death. In this system, each fly was put in its own glass tube (25 mm diameter, 125 mm length), where three ray rings of an infrared light monitor at three different planes were placed (close to the two ends of the tube and one in the middle). The activity of each tube was measured every minute as the number of times the fly passed through an infrared beam. An agar‐based gel diet consisting of sugar, yeast, hydrolysate, agar, nipagin and water (4:1:0.2:0.1:20) was prepared to provide adults with both nutrients and water simultaneously. We used three different gel diets which differed in the sugar and yeast hydrolysate content in the gel (50%, 20%, and 10%, represented by treatment C‐50, C‐20, and C‐10 accordingly). The gel diet was replaced every 4 days to avoid dehydration. Females' mortality was recorded daily with visual observations until the death of all individuals.

In our analysis, individual activities from the record of the middle monitor were used and aggregated per hour for a total number of 96 tubes under three different treatment levels (C‐10, C‐20, C‐50). Each treatment level corresponds to 32 tubes. See Figures S1, S2, and S3 in the supplement for the daily activities under each treatment along with individual lifespans.

## 3 STATISTICAL METHODS

### 3.1 Function‐valued stochastic process

Denote the time coordinate of the calendar days by t∈T and the repeated measurement coordinate of hours within a day by s∈S. Let $X\left(s,t\right)$ to be the function‐valued stochastic process representing the activity level at day *t* and hour *s*. Mean function and the covariance function of the process can be defined as

(1) $$\mu \left(s,t\right)=E\left(X\left(s,t\right)\right),$$

$$G\left(\left(s_{1},t_{1}\right),\left(s_{2},t_{2}\right)\right)=E\left(X\left(s_{1},t_{1}\right)X\left(s_{2},t_{2}\right)\right)$$

$$\;-\mu \left(s_{1},t_{1}\right)\mu \left(s_{2},t_{2}\right),s_{1},s_{2}\in S,t_{1},t_{2}\in T.$$

Mercer's theorem implies the spectral decomposition of the covariance surface G as follows,

$$G\left(\left(s_{1},t_{1}\right),\left(s_{2},t_{2}\right)\right)=\sum_{k=1}^{\infty }\lambda_{k}\gamma_{k}\left(s_{1},t_{1}\right)\gamma_{k}\left(s_{2},t_{2}\right),$$

where $\lambda_{k}$ and $\gamma_{k}$ are eigenvalues and two‐dimensional eigenfunctions of the covariance operator with covariance kernel G. By the Karhunen‐Loève expansion (Karhunen, 1946) one can represent $X\left(s,t\right)$ through two‐dimensional Functional Principal Component Analysis (FPCA), a key technique of FDA, as

(2) $$X\left(s,t\right)=\mu \left(s,t\right)+\sum_{k=1}^{\infty }\xi_{k}\gamma_{k}\left(s,t\right),$$

where $\xi_{k}=\int_{T}\int_{S}\left(X\left(s,t\right)-\mu \left(s,t\right)\right)\gamma_{k}\left(s,t\right)\text{dsdt}$ are the principal components of the process. These components are zero mean uncorrelated random variables representing the fluctuations of the process $X\left(s,t\right)$ around the mean function $\mu \left(s,t\right)$. In practice, one works with the first K components and the approximated process $X\;\left(s,t\right)=\mu \left(s,t\right)+\sum_{k=1}^{K}\;\xi_{k}\gamma_{k}\left(s,t\right).$ We choose K=3 in the following analysis.

Modes of variation (Castro et al., 1986) are a common tool to interpret and visualize the components of FPCA. They focus on the contributions of each eigenfunction to the stochastic behavior of the underlying process. The k‐th mode of variation is a set of functions indexed by a parameter α∈ℝ given by

(3) $$m_{k}\left(s,t,\alpha \right)=\mu \left(s,t\right)+\alpha \sqrt{\lambda_{k}}\gamma_{k}\left(s,t\right),\;\;\alpha \in \left(-\infty ,\infty \right).$$

### 3.2 Prediction of remaining lifetime

Previous approaches for remaining lifetime prediction (Müller & Zhang, 2005) need to be adjusted for the scenario of a function‐valued rather than scalar‐valued activity process that we adopt here. We denote the lifetime or age‐at‐death of a subject by T and assume that for each subject the daily activity X is recorded continuously. We denote the observed activity trajectory for an individual that is still alive at time $T_{0}$ by $\tilde{X}\left(s,t,T_{0}\right),0\leq t\leq T_{0},s\in S,$ that is

$$\tilde{X}\left(s,t,T_{0}\right)=\left\{\begin{array}{c} X\left(s,t\right)\;\;\text{if}\;\;\;T_{0}\leq T,0\leq t\leq T_{0},\\ \text{unobserved}\;\;\;\;\;\;\text{if}\;\;T_{0}>T. \end{array}\right.$$

Our goal is to predict the remaining lifetime $T-T_{0},$ given $\tilde{X}\left(s,t,T_{0}\right).$ The activity record thus is censored by the age‐at‐death of a fly, and there is an inherent trade‐off between the selected age span and the number of flies whose activity can be observed over their entire lifespan. Our approach is based on nonparametric data analysis and thus is very flexible. If one is willing to sacrifice some of this flexibility, some more complex approaches to deal with this censoring issue are provided by joint modeling (Ding & Wang, 2008; Müller & Zhang, 2005). Denote the expected remaining lifetime conditional on $\tilde{X}\;\;$ as $r_{\tilde{X}}\left(T_{0}\right)$, i.e.,

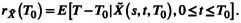




The functional linear regression model (Müller, 2005; Müller & Stadtmüller, 2005; Ramsay & Silverman, 2005) is given by

$$r_{\tilde{X}}\;\left(T_{0}\right)=r_{0}\left(T_{0}\right)$$

(4)

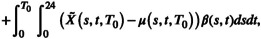

where $r_{0}\left(T_{0}\right)$ is a nonrandom intercept function, $\beta \left(s,t\right)$ is the smooth coefficient surface and $\mu \left(s,t,T_{0}\right)$ is the mean function of $\tilde{X}\left(s,t,T_{0}\right).$ The functional linear regression model can be equivalently rewritten as

$$T-T_{0}=r_{0}\left(T_{0}\right)$$

(5)

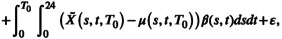

where ε is a zero mean finite variance random error. If one represents the coefficient surface $\beta \left(s,t\right)$ using the orthonormal eigenbasis $\gamma_{k}$ from equation (2), such that $\beta \left(s,t\right)=\sum_{k=1}^{\infty }\beta_{k}\gamma_{k}\left(s,t\right)$, then the model for the mean remaining lifetime becomes

(6) $$r_{\tilde{X}}\;\left(T_{0}\right)=r_{0}\left(T_{0}\right)+\sum_{k=1}^{\infty }\beta_{k}\xi_{k},$$

where the $\xi_{k}$ are the principal components as in equation (2).

In order to assess the strength of the association, a pseudo‐*R* square measure can be used that is defined as

$$R^{2}=1-\frac{\mathrm{Var}\left(\varepsilon \right)}{\mathrm{Var}\left(T-T_{0}\right)},$$

where larger values of $R^{2}$ indicate that the predictor explains more of the variability of the response, i.e., of remaining lifetime.

### 3.3 Conditional distributions of remaining lifetime

The conditional distribution of remaining lifetime $T-T_{0}$ at a current time $T_{0}$ of a subject, given an observed trajectory $\tilde{X}$ up to time $T_{0}$, is defined as 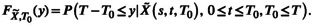 Müller and Zhang (2005) proposed to construct the conditional distribution function of the remaining lifetime by the further assumption that the linear predictor function $\eta \left(T_{0}\right)=\int_{0}^{T_{0}}\int_{0}^{24}\tilde{X}\left(s,t,T_{0}\right)\beta \left(s,t\right)\text{dsdt}$ determines the conditional distribution,

(7) $$\begin{array}{c} F_{\tilde{X},T_{0}}\left(y\right)=P\left(T-T_{0}\leq y|\tilde{X}\left(s,t,T_{0}\right)\right)\\ \;=P\left(T-T_{0}\leq y|\eta \left(T_{0}\right)\right)\\ \;=\varphi_{T_{0},y}\left\{\eta \left(T_{0}\right)\right\}. \end{array}$$

Here the unknown function $\varphi_{T_{0},y}\left(\;\cdot \;\right)$ is assumed to be smooth in both y and $T_{0}$. Estimating conditional remaining lifetime distributions then is equivalent to estimating functions $\varphi_{T_{0},y}\left\{\;\cdot \;\right\}$ and $\eta \left(\;\cdot \;\right)$ and nonparametric smooth estimators employing boundary kernels (Müller and Wang (1994)) can be utilized to estimate the smooth function $\varphi \left(\;\cdot \;\right).$ See Section 3.3 in Müller and Zhang (2005) for more details.

## 4 RESULTS

### 4.1 Early‐age activity profile and connection to diet

**FIGURE 1:**
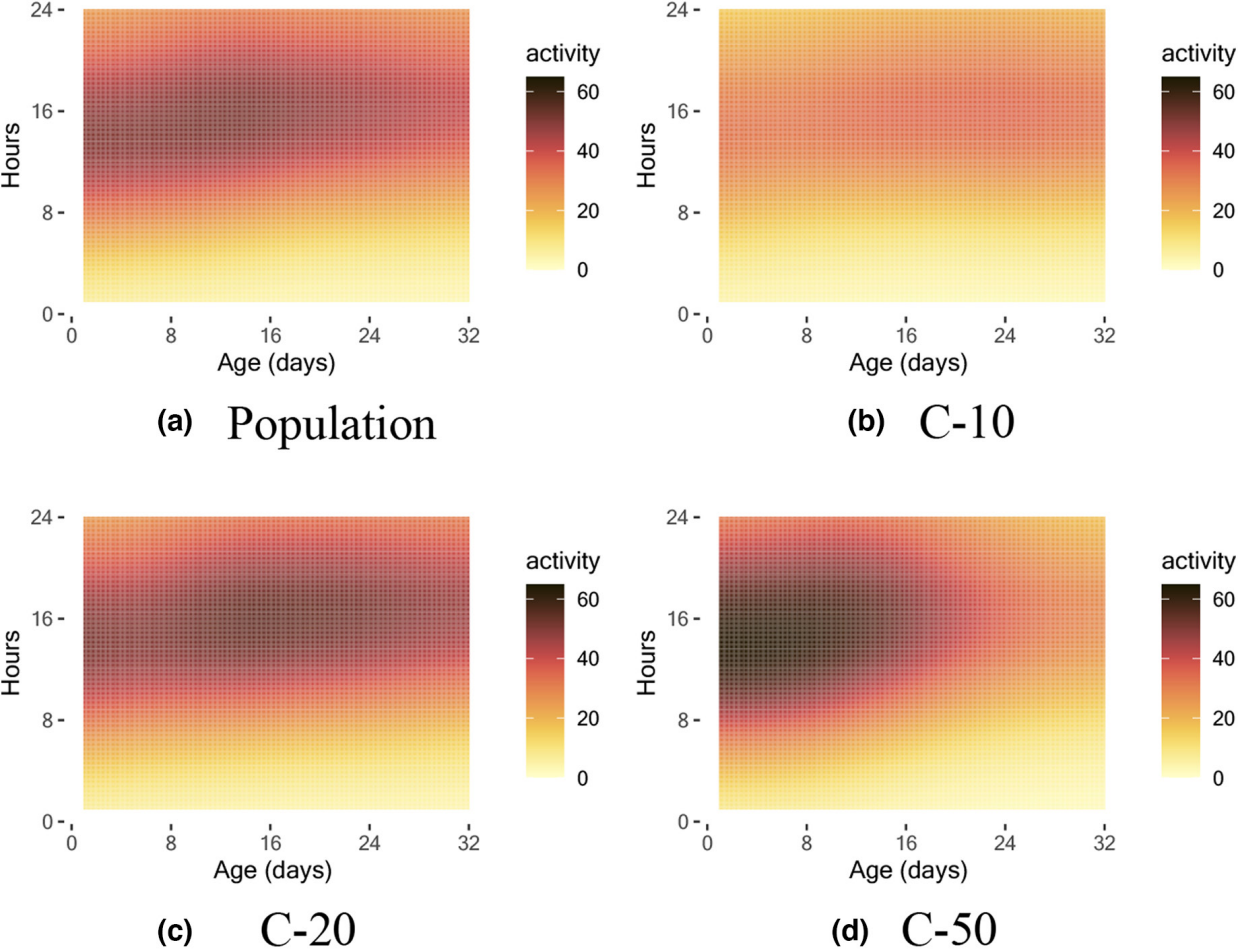
Estimated mean functions of early‐age activity, as per (1) of Section 3.1, for population level and three diet levels C‐10, C‐20, and C‐50.

The estimated mean process as per (1) of Section 3.1 is shown in Figure 1, along with the mean process under different diet levels. During a 24‐h cycle, medflies are most active and energetic during periods of illumination as indicated by the light sensor (07:00–21:00) and less active during periods of darkness. With regard to activity dynamics, older medflies exhibit a tendency to rise later. A low nutrition level (C‐10) is associated with a relatively delayed activity peak, while a high nutrition level (C‐50) is linked to an earlier activity peak.

**FIGURE 2:**
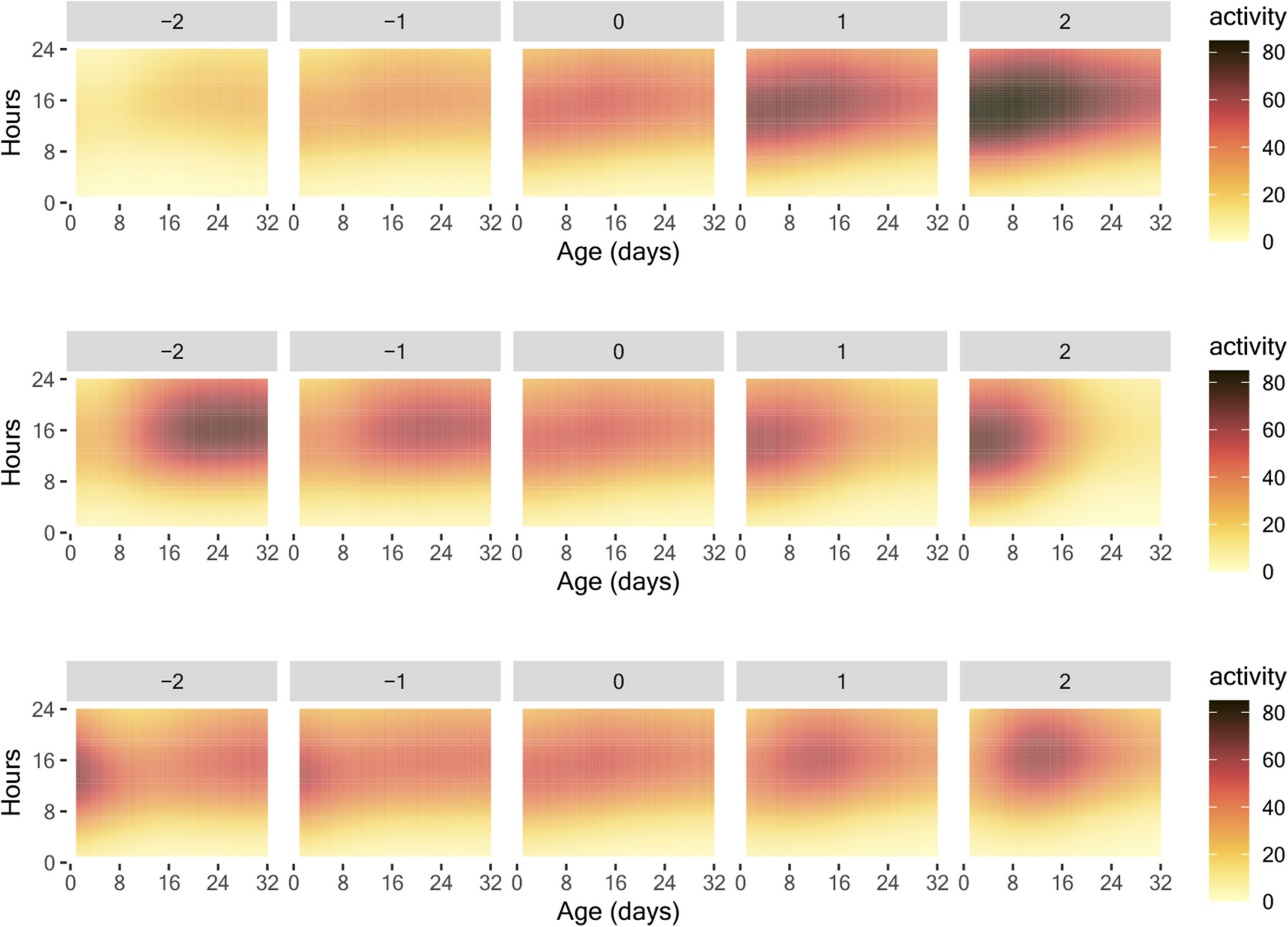
The modes of variation, as per (3) in Section 3.1, vary with the parameter α ranging from −2 to 2 (left to right). The three rows correspond to the modes of variation of the first three two‐dimensional eigenfunctions. The eigenfunctions are illustrated in Figure S5 in the Appendix.

Over 30% of the variation in the early‐age activity model (2) is explained by the first three eigenfunctions, as depicted in Figure S5 in the supplement. The modes of variation of the first three eigenfunctions as per (3) in Section 3.1 are illustrated in Figure 2 to facilitate the interpretation of the eigenfunctions. The predominant variation is reflected in the contrasting patterns of the activity between early and late days and between daytime and nighttime. This indicates that the predominant source of variation is attributed to young medflies during their active daytime hours. The second eigenfunction highlights a contrasting pattern between early and late ages, while the third eigenfunction focuses on the contrast between the activity during day 8 to day 20 (daytime only) and the other periods.

To further investigate the treatment effect on early‐age medfly activity, we fit linear regression models using scores of the FPCA as the response variables and treatment levels as predictors. Table 1 shows the summarized fitted results. To enhance the visualization of the treatment effect, we use pairwise score representations in Figure S4 in the supplement to facilitate interpretation of the regression relation.

**TABLE 1:** The results of the linear regression using the FPCA scores of the early‐age activity as the response variables (top to bottom) and treatment levels as predictors (left to right).

Response	Intercept	C‐20	C‐50	$R^{2}$
$\xi_{1}$	**−243.29 (<0.001)**	**295.37 (<0.001)**	**389.43 (<0.001)**	0.23
$\xi_{2}$	**−132.30 (0.01)**	50.43 (0.455)	**321.96 (<0.001)**	0.28
$\xi_{3}$	−50.03 (0.24)	27.53 (0.63)	**13.31 (0.05)**	0.06

*Note*: The corresponding *p*‐values are included in brackets and the $R^{2}$ values are listed in the final column. Coefficients with significant *p*‐values (*p*‐value≤0.05) are shown in bold.

Consistent with the results of the mean activity profiles, a low nutrition level (C10) is associated with a diminished contrast between daytime and nighttime activity and a relatively late peak in activity. On the other hand, medium nutrition (C‐20) and high nutrition (C‐50) result in a relatively early peak in activity and an increased contrast between daytime and nighttime activity, with high nutrition (C‐50) having a strong connection to high early‐age activity.

### 4.2 Relationship between early‐age activity and lifespan

The preceding section focused on the early‐age activity profile of medflies, while here we explore the relationship between the activity profile and longevity and demonstrate that the activity profile can be used as a biomarker for longevity.

We present the results of the functional linear regression model (4) with treatment levels and score representations of the early‐age activity profile as predictors. By using the two‐dimensional orthonormal eigenfunctions in the FPCA, the regression model can be reformulated through equation (6), and the results for the fitted model are summarized in Table 2 where $R^{2}$ = 0.21. We also provide the regression model under each treatment level in Table S1 in the Supplement. A medium nutrition diet (C‐20) is associated with longer lifespans compared with the other treatments. However, a higher score for the eigenfunction $\gamma_{1}$ (representing high levels of activity in early age and a large contrast between daytime and nighttime activity) can be associated with increased mortality risk. This effect is particularly pronounced for individuals under a high nutrition diet (C‐50) as they may have exhausted themselves during their early life or daytime periods. These results indicate that the medfly activity profile can serve as a biomarker of longevity.

**TABLE 2:** The regression coefficients along with 95% confidence intervals for functional linear regression as per model (6) in Section 3.2 where significant coefficients are bold.

Coefficient	Fitted result	Confidence interval
$r_{0}\left(T_{0}\right)$	**18.606**	**(8.502, 27.811)**
$\beta_{1}$	**−0.013**	**(−0.034, 0.001)**
$\beta_{2}$	−0.013	(−0.030, 0.002)
$\beta_{3}$	−0.005	(−0.025, 0.011)
$C_{20}$	**11.856**	**(0.218, 19.757)**
$C_{50}$	8.726	(−6.044, 26.473)

*Note*: Score representations of the early‐age activity profile in Section 4.1 together with treatment levels are the predictors, while the remaining lifespan is the response.

**FIGURE 3:**
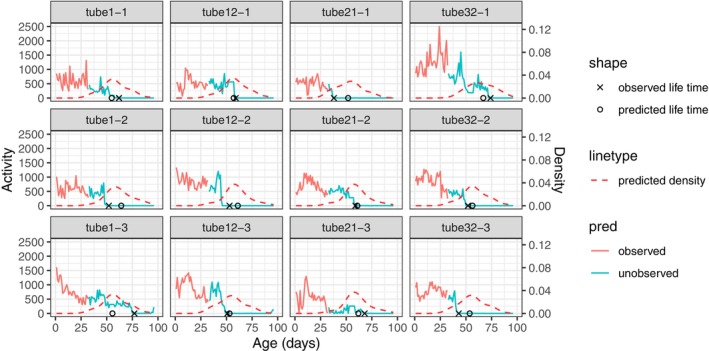
The prediction of the remaining lifespan as per model (6) in Section 3.2, where the score representations of the early‐age activity profile in Section 4.1 together with treatment levels are predictors, while the remaining lifespan is the response. The figure displays three rows, corresponding to the randomly selected tubes under different treatment levels (C‐10, C‐20, and C‐50, top to bottom). The observed age‐at‐death is marked as a cross while the predicted age‐at‐death is marked as a circle for each tube. The predicted conditional density of age‐at‐death as per model (7) in Section 3.3 is shown as red dashed line for each tube.

Figure 3 presents the daily activity curves along with the predicted lifespan in a sample of randomly selected tubes. The results suggest that the prediction of lifespan is in good agreement with the actual age‐at‐death. The conditional density curves show the predicted distribution of age‐at‐death, with higher values indicating a higher probability of death at that time.

### 4.3 Before‐death activity profile

To study the activity patterns of medflies before they die, we model the before‐death activity as a function‐valued stochastic process. The time coordinate is thanatological age represented by thanatological days (days going backwards from the day of death), while the repeated measurement coordinate remains unchanged and corresponds to the hour within a day. The mean function and eigenfunctions can be defined and estimated similarly as in Section 3.1.

**FIGURE 4:**
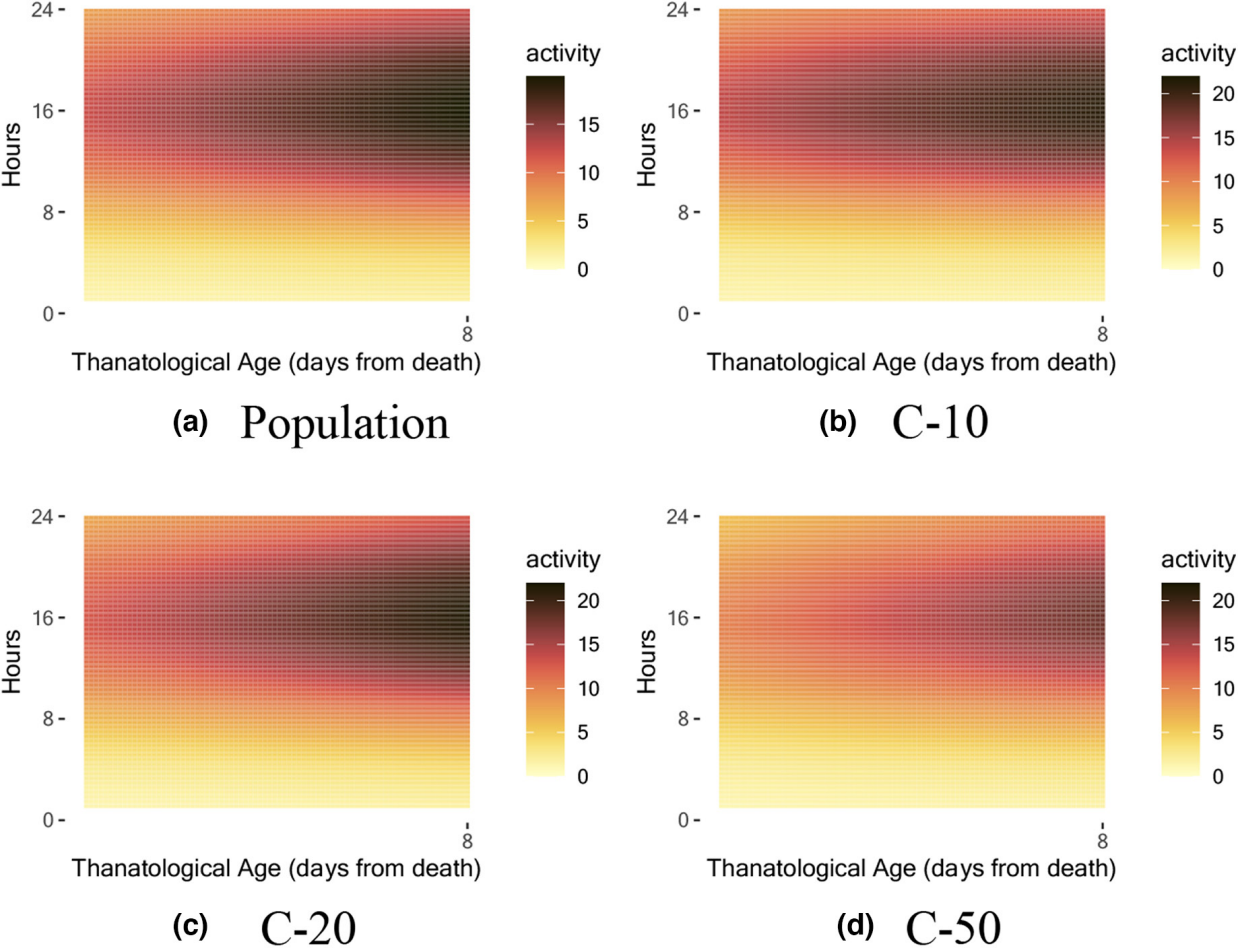
Estimated mean functions of before‐death activity profile, as per (1) of Section 3.1, for (a) population level and three diet levels (b, c, and d) C‐10, C‐20, and C‐50.

Figure 4a shows the population level mean function of the medfly activity modeled in the thanatological age scale, where the time coordinate starts from the day of death and the hour within the day remains unchanged. The mean function displays the average activity pattern of the medflies, indicating that they tend to be more active during the hours when the light sensor is on (7:00–21:00) and less active during the hours when the light sensor is off. Additionally, the mean function shows a decline in activity as the medflies approach their death.

**FIGURE 5:**
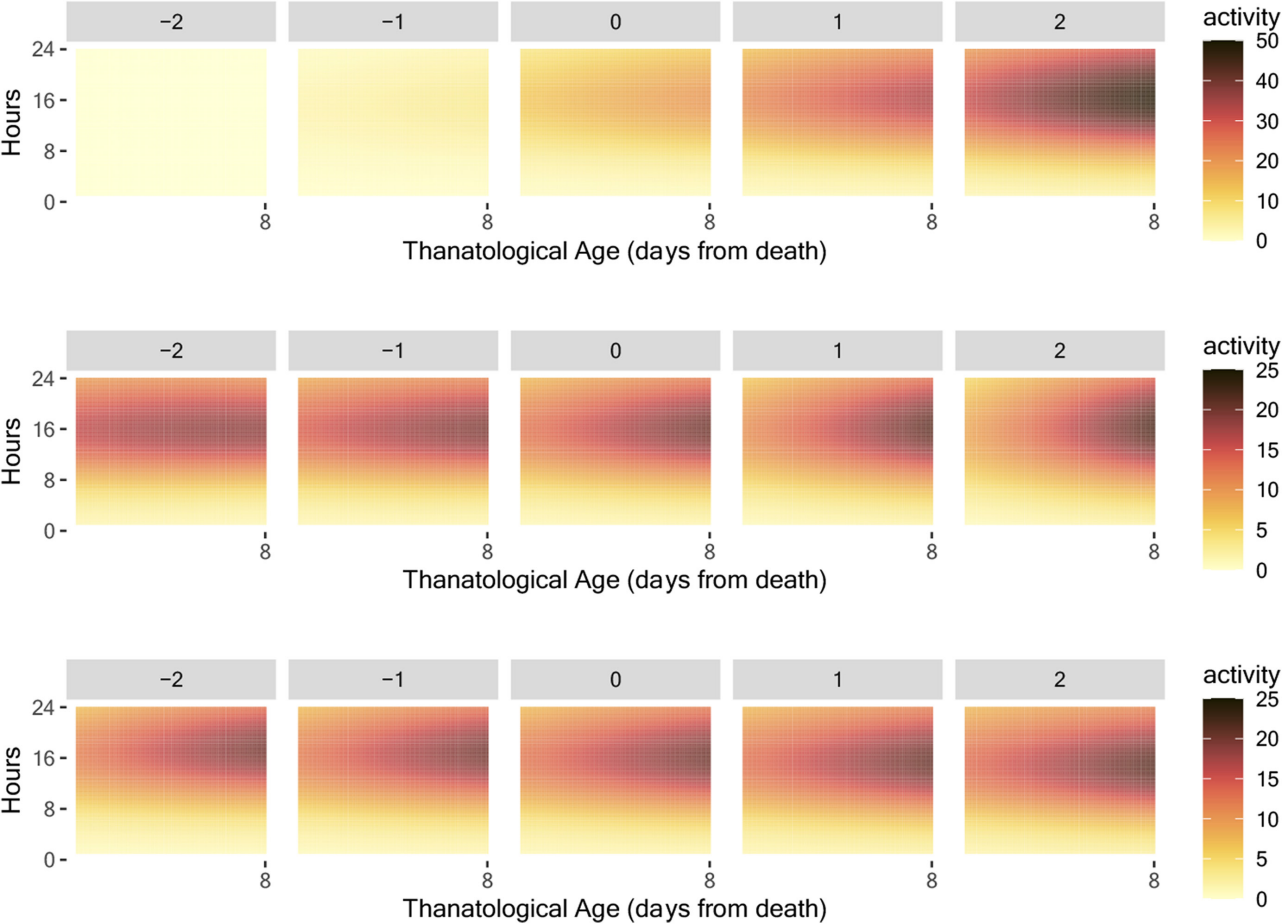
The modes of variation, as per (3) in Section 3.1, vary with the parameter α ranging from −2 to 2 (left to right). The three rows correspond to the modes of variation of the first three two‐dimensional eigenfunctions. The eigenfunctions are illustrated in Figure S6 in the Appendix.

Over 50% of the variation in the before‐death activity is explained by the first three two‐dimensional eigenfunctions, as depicted in Figure S6 in the supplement. Modes of variation of the first three eigenfunctions as per (3) in Section 3.1 are illustrated in Figure 5. The primary variation can be seen in the decline of activity during the daytime leading up to death. The second eigenfunction emphasizes the level of activity prior to death. The third eigenfunction concentrates on the decrease in activity during the early morning hours.

**FIGURE 6:**
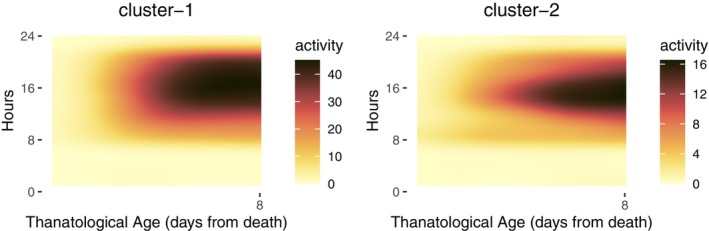
Representatives of the two clusters identified for the activity profiles before death—abrupt end of the activity (left) and dwindling of activity (right).

The FPCA scores associated with the first three eigenfunctions are utilized for downstream cluster analysis. We employ a Gaussian mixture model with a diagonal covariance matrix to identify two underlying clusters, as shown in Figure 6. The first cluster can be connected with the sudden death feature of the medflies, while the second cluster reflects a dwindling pattern of activity as medflies approach their death.

To gain a deeper understanding of the differences between the two clusters, we randomly select six flies from each cluster and present their activity data in Figures S7 and S8 in the supplement. For cluster 1, four tubes (24–3, 15–2, 10–1, and 2–2) exhibit a noticeable decline in activity just before death. For cluster 2, all six tubes demonstrate a gradual decrease in activity leading up to death.

## 5 DISCUSSION

We model the daily activity of the Mediterranean fruit fly as a functional‐valued stochastic process, taking into account the repeated measurements of activity for each day during their lifespan and using advanced FDA methodology. The longitudinal time is represented by the calendar day, and the repeated observations are the recorded times within each day. FPCA is used to effectively demonstrate the modes of variation of the process.

The study identified three distinct patterns of variation in the early‐age activity profile and explored their connection to diet. A low nutrition level (C‐10) is associated with a diminished contrast between daytime and nighttime activity and a relatively late peak in activity. On the other hand, medium nutrition (C‐20) and high nutrition (C‐50) result in a relatively early peak in activity and an increased contrast between daytime and nighttime activity, with high nutrition (C‐50) having a strong connection to high early‐age activity.

Our analysis also indicates that there is a strong connection between daily activity in early age and subsequent mortality and demonstrates that the activity profiles can serve as a biomarker. We apply a functional linear regression model that uses the activity within the first 32 days and the treatment received by the medfly to predict its lifespan. Increased mortality risk is linked with higher activity levels and greater contrast between daytime and nighttime activity, particularly in the high nutrition level group (C‐50). Individuals receiving medium nutrition (C‐20) are predicted to have a longer lifespan compared to those in the low nutrition group (C‐10) and high nutrition group (C‐50). We also present predicted lifespan distributions for individual subjects, where a higher value of the density function of the predicted distribution indicates a higher probability of death for the medfly. All of this shows that activity profiles can serve as a useful biomarker of longevity.

It is important to note that the proposed methodology can be generally used for tracking medfly activity across various conditions and also for other species as long as continuous recording of activity is available over the lifespan of the respective species. Furthermore, it is essential to acknowledge that our current findings stem from observing the activity of unmated female adult medflies living in an isolated glass tube. The cost of reproduction has been strongly correlated with the aging process in medflies' life history traits in the past (Chapman et al., 1998). Therefore, in future studies, it would be extremely interesting to investigate the differences between the locomotory activity patterns for virgin and mated flies and their relationship with aging. In addition, the social interactions and crowding that happened in the field could potentially impact activity profiles and lifespan (Gaskin et al., 2002; Warburg & Yuval, 1997), although tracking these effects can pose significant challenges.

Additionally, exemplifying the adaptability of the proposed approach, we demonstrate that the technique to model the early‐age activity profile can also be applied to the modeling of before‐death activity profiles and provides insights into the before‐death activity. The use of a Gaussian mixture model cluster analysis identifies two distinct patterns in the before‐death activity profiles, allowing for a deeper understanding of the process leading up to death. One cluster is characterized by a slow decline in daily activity and the other is connected with a sudden activity decline before death. Understanding these modes of dying is of general interest to gain further insights into longevity.

Fruit flies, as fully metamorphosed insects, undergo a life cycle encompassing four distinct stages: egg, larva, pupa, and adult. They infest fruits by depositing their eggs beneath the fruit's surface, where the larvae subsequently feed on the fruit's interior. Upon reaching maturity, these larvae emerge from the fruit and undergo pupation in the soil. Given the considerable challenges in observing larval locomotor activity within natural fruit environments and artificial food mediums (Bittern et al., 2022; Risse et al., 2017), our study centers on observing and analyzing the locomotor activity of adult flies. Although numerous studies link immature experiences, such as rearing at lower temperatures or consuming protein‐deficient diets, with adult fitness, reproductive success, and longevity, further investigation is required to ascertain the precise impact of immature experiences on adult aging dynamics (Yadav & Sharma, 2014). As mentioned above, fruit flies are holometabolous insects, and they are commonly used in aging studies. Interestingly, studies in the past argued that holometabolous species buffered adult phenotypes from immature developmental variation, thus hemimetabolous insects may be more comparable with vertebrates when focusing on aging studies (Archer & Hunt, 2015; Lyn et al., 2011). From this point of view, it would be interesting to apply our models to hemimetabolous species to characterize the aging process in plenty of different types of organisms, as the LAM system has been used in the past for imperfectly metamorphosed species (Kaniewska et al., 2020). As an extension, exploration of the connections of underlying molecular and physiological mechanisms (Goossens et al., 2020) and activity profiles would aid to gain a better understanding of the factors governing activity.

## AUTHOR CONTRIBUTIONS

Han Chen (Methodology, Software, Formal analysis, Writing—original draft). Hans‐Georg Müller (Conceptualization, Methodology, Software, Formal analysis, Resources, Writing—original draft, Supervision). Vassilis Rodovitis (Experimental design, Data curation, Writing—original draft). Nikos Papadopoulos (Experimental design, Data curation, Supervision). James R. Carey (Conceptualization, Resources, Writing—original draft, Data curation, Supervision, Funding acquisition).

## FUNDING INFORMATION

No funding information provided.

## CONFLICT OF INTEREST STATEMENT

The authors declare no competing interests.

## Supporting information


Appendix S1.

## Data Availability

The data supporting this study's findings are available from James R. Carey upon reasonable request (email address: jrcarey@ucdavis.edu).
