# Supplementary material for: Daily activity profiles over the lifespan of female medflies as biomarkers of aging and longevity
Source: Aging Cell. 2024 Jan 24;23(4):e14080. doi: 10.1111/acel.14080 (PMC11019124; doi:10.1111/acel.14080)
Supplement: Supplementary file 1 — Appendix S1. [file ACEL-23-e14080-s001.docx]

Supplement: Daily activity profiles over the lifespan of female medflies as biomarkers of aging and longevity

Han Chen^1^, Hans-Georg Müller^1, *^, Vasilis G. Rodovitis^2^, Nikos T. Papadopoulos^2^, James R. Carey^3^

^1^Department of Statistics, University of California at Davis, Davis, CA, 95616, USA

^2^Department of Agriculture Crop Production and Rural Environment, University of Thessaly, Volos, 38446, Greece

^3^Department of Entomology, University of California at Davis, Davis, CA, 95616, USA

^*^Corresponding author. Email address: hgmueller@ucdavis.edu

S1. Daily Activity of the Medfly

Figure A.1: Daily medfly activity under C-10 treatment. The black curves correspond to the raw daily activity trajectory and the blue curves correspond to the local linear smoothing trajectory using Epanechnikov kernel and bandwidth 3.

Figure A.2: Daily medfly activity under C-20 treatment. The black curves correspond to the raw daily activity trajectory and the blue curves correspond to the local linear smoothing trajectory using Epanechnikov kernel and bandwidth 3.

Figure A.3: Daily medfly activity under C-50 treatment. The black curves correspond to the raw daily activity trajectory and the blue curves correspond to the local linear smoothing trajectory using Epanechnikov kernel and bandwidth 3.

S2. Activity Profile Scores

Figure A.4: Pair plots of scores of the FPCA of the early age activity in Section 4.1 with the color legend indicates different treatment levels.

S3. Additional Regression Model

Table A.1: The regression coefficients under different treatment levels (left to right) as per model (6) in Section 3.2 with score representations of early-age activity profiles in Section 4.1 as predictors (top to bottom) and the remaining lifetime as the response. The $p$-values for each coefficient are also given in the bracket and the significant coefficients ($p$-value$<= 0.05$) are displayed in bold font.

| Coefficient | C-10 | C-20 | C-50 |
| --- | --- | --- | --- |
| $r_{0}\left( T_{0} \right)$  $\beta_{1}$  $\beta_{2}$  $\beta_{3}$ | **17.344 (<0.01)**  -0.016 (0.39)  -0.029 (0.18)  0.017 (0.63) | **31.828 (<0.01)**  0.006 (0.52)  0.014 (0.40)  0.003 (0.72) | **30.975 (<0.01)**  **-0.029 (0.01)**  -0.008 (0.55)  **-0.028 (0.05)** |

S4. Eigenfunctions

Figure A.5: The first three eigenfunctions for FPCA of early age medfly activity as per (2) of Section 3.1. The $Y$-axis represents the coordinate of hours and the $X$-axis represents the coordinate of days from birth. The number in the bracket corresponding to each eigenfunction indicates the fraction of variance explained. Modes of variation for each eigenfunction are illustrated in Figure 2.

Figure A.6: The first three eigenfunctions for FPCA of early age medfly activity as per (2) of Section 3.1. The $Y$-axis represents the coordinate of hours and the $X$-axis represents the coordinate of days from birth. The number in the bracket corresponding to each eigenfunction indicates the fraction of variance explained. Modes of variation for each eigenfunction are illustrated in Figure 5.

S5. Cluster Analysis

Figure A.7: Randomly selected tubes corresponding to cluster 1 as in Section 4.3.

Figure A.8: Randomly selected tubes corresponding to cluster 1 as in Section 4.3.
